# The exploration of risk factors of concurrent bacteraemia in patients critically ill with severe dengue

**DOI:** 10.1099/jmm.0.000388

**Published:** 2016-12-16

**Authors:** Chin-Ming Chen, Khee-Siang Chan, Kuo-Chen Cheng, Willy Chou, Hui-Chun Chao, Chiu-Yin Yeh, Wen-Liang Yu

**Affiliations:** ^1^​ Department of Recreation and Health-Care Management, Chia Nan University of Pharmacy and Science, Tainan, Taiwan, ROC; ^2^​ Department of Intensive Care Medicine, Chi-Mei Medical Center, Tainan, Taiwan, ROC; ^3^​ Department of Internal Medicine, Chi-Mei Medical Center, Tainan, Taiwan, ROC; ^4^​ Department of Safety Health and Environment, Chung Hwa University of Medical Technology, Tainan, Taiwan, ROC; ^5^​ Department of Medicine, Taipei Medical University, Taipei, Taiwan, ROC

**Keywords:** concurrent bacteraemia, severe dengue, intensive care unit, risk factor

## Abstract

We investigated the clinical features of intensive care unit (ICU) patients with concomitant severe dengue infection and bacteraemia to identify risk factors for this comorbidity. The records of all ICU dengue patients admitted during the period of 31 July–30 November 2015 were reviewed. Patients with ‘concurrent bacteremia’ (positive bacterial blood culture within 72 h of ICU admission) were identified. ICU admission was required for 142 patients, of which 22 (15.5 %) had concurrent bacteraemia. Species of the genus *Streptococcus* was the most common pathogens, followed by *Escherichia coli* then species of the genus *Staphylococcus*. Patients with a severe dengue infection and bacteraemia had higher APACHE II and TISS scores, C-reactive protein (CRP) levels and leukocyte counts, positive fluid balances, longer activated partial thromboplastin times (APTTs), higher lactate levels and more kidney failure, but controls (severe dengue patients without bacteraemia) had higher Glasgow Coma Scale (GCS) scores, higher albumin levels and more abdominal pain (all *P*<0.05). Patients with bacteraemia had a higher mortality rate than did ontrols (40.9 vs 18.3 %; *P*=0.018). Multiple logistic regression analysis showed that bacteraemia was significantly positively associated with the following independent predictors: higher CRP levels [adjusted odds ratio (aOR): 1.026; 95 % confidence interval (CI): 1.008–1.044; *P*=0.005], and longer APTTs (aOR: 1.034; 95 CI: 1.004–1.065; *P*=0.027). Concurrent bacteraemia is not uncommon in severe dengue patients in the ICU, and it is associated with high mortality. Higher CRP levels and longer APTTs were two independent risk factors associated with bacteraemia.

## Introduction

Dengue is a common arthropod-borne virual (arbovirus) disease in tropical and subtropical regions (Horstick *et al.*, 2015; Stanaway *et al.*, 2016). Based on the World Health Organization’s estimation, the annual incidence of dengue infection ranges from 50 million to 200 million (Murray *et al.*, 2013). However, the true number of dengue infections is probably much higher because of a lack of adequate global surveillance of the disease and because it is easy to misdiagnose. The initial symptoms and signs of dengue disease are nonspecific, and fever is one of the most common clinical manifestations of dengue infections (Simmons *et al.*, 2012). The clinical presentations of dengue infections can be asymptomatic or mild, or severe in cases that require admission to the intensive care unit (ICU) (Guzman & Harris, 2015).

In the ICU, a concomitant bacterial infection is not uncommon in patients with severe dengue infections. Amâncio *et al.* (2015) reported that, of 97 dengue patients admitted to the, ICU, 45 (46.4 %) were treated with antibiotics for bacterial infections and seven (7.2 %) died because of concomitant bacterial infections. The clinical manifestations of dengue and bacterial infections may overlap; therefore, clinicians might overlook the possibility of concurrent bacteraemia in a dengue-endemic setting. Most importantly, if the concomitant bacteraemia is not quickly diagnosed and additional appropriate antibiotic treatment is not prescribed, the affected patients will be at great risk of dying. However, it is difficult to determine which subgroups of dengue patients have concomitant bacterial infections, and for clinicians to know which patients need what antibiotics, and when they need them. Although several studies (Chai *et al.*, 2007; Lee *et al.*, 2005; Premaratna *et al.*, 2015; Thein *et al.*, 2015) have reported the clinical characteristics and risk factors of concurrent bacteraemia in adult patients with dengue infections, none of these focused on patients with severe dengue who required admission to the ICU. Therefore, we investigated the clinical features of patients with concomitant severe dengue infection and bacteraemia, in order to identify possible risk factors for acquiring a severe dengue/bacteraemia co-infection.

## Methods

### Patients and hospital setting.

This was a retrospective cohort study of all patients on the 2015 dengue registry of Chi Mei Medical Center, a tertiary referral hospital with 96 adult ICU beds. All of the patients with laboratory-confirmed dengue infection were included if they matched at least one of the five following positive laboratory results: nonstructural protein 1 (NS1) antigen test, dengue immunoglobulin-M (IgM), dengue immunoglobulin-G (IgG), dengue polymerase chain reaction (PCR) or viral isolation. According to the WHO (2009) criteria (Hadinegoro, 2012; WHO, 2009), patients were classified as having dengue with warning signs if there were reports of abdominal pain or tenderness, vomiting, clinical fluid accumulation, mucosal bleeding, lethargy or restlessness, hepatomegaly and a rise in hematocrit concurrent with a rapid drop in platelet count. Severe dengue (group C) criteria included one or more of the following symptoms: (1) severe plasma leakage leading to shock or fluid accumulation with respiratory distress, (2) severe bleeding, (3) severe organ involvement and (4) transaminase levels >1000 IU l^−1^. Between 31 July and 30 November 2015, the major outbreak period, 4787 patients were diagnosed with dengue infections but only 142 matched the severe dengue criteria and were initially admitted to the ICU from the emergency room (ER); other patients were transferred from general wards if their disease progressed to severe after admission.

‘Concurrent bacteraemia’ was defined as a positive bacterial blood culture within 72 h of a patient’s ICU admission. We excluded patients with a positive culture after 72 h because it was not possible to determine whether their bacteraemia was concurrent with the dengue infection or was nosocomial. We also excluded those with indeterminate bacteraemia when only one of their two sets of blood cultures was positive for skin flora. We first compared the clinical data between severe dengue patients with and without bacteraemia. We then compared severely ill dengue patients with bacteraemia to a control group of age-, gender-, Acute Physiology and Chronic Health Evaluation II (APACHE II) score-, Therapeutic Intervention Scoring System (TISS) score-, and Glasgow Coma Scale (GCS) score-matched patients with bacteraemia but no dengue infection (1 : 2 ratio). The data were collected in routine clinical practice and the analyses were done retrospectively; therefore, informed consent was waived. The study was approved by the Institutional Review Board of Chi Mei Medical Center (IRB no. 10503–005) (See Tables 1, 2 and 3).

### Blood culture.

Blood was inoculated into BACTEC culture bottles using the BACTEC 9240 Blood Culture System (Becton Dickinson). All bacterial strains were identified to the species level using conventional methods and were verified using the API-20E System (BioMérieux Vitek) or the Vitek 2 ID-GNB identification card (BioMérieux).

### Variables measured.

The patients’ demographic data and APACHE II, TISS and GCS severity scores at ICU admission were recorded. Information about underlying comorbidities, initially presented symptoms, clinical variables, laboratory data and therapeutic intervention during admission were collected for comparison. See Appendix 1 (available in the online Supplementary Material) for other definitions.

### Statistical analysis.

Continuous and categorical variables are expressed as means±sd, or as frequencies and percentages, as indicated. Univariate analysis with Student’s *t-*test, Wilcoxon rank-sum test, χ^2^ or Fisher exact tests was used to examine the differences between dengue groups with and without concurrent bacteraemia. Multivariate analysis was also used to analyse the risk factors for dengue patients with primary bacteraemia by concurrently entering all of the demographic, clinical and laboratory variables into the logistic regression model. Significance was set at *P*<0.05. Adjusted odds ratios (aORs) and 95 % confidence intervals (CIs) were also calculated. SPSS 19.0 for Windows (SPSS) was used for all data analyses.

## Results

During the study period, 142 patients (mean age: 70.0 years) with severe dengue required ICU admission. Their mean severity scores were APACHE II=18.0, TISS=22.9 and GCS=12.0. The two most common comorbidities were hypertension [*n*=90 (63.4 %)] and diabetes mellitus [*n*=70 (49.3 %)]. The haematological system failed most frequently [*n*=112 (78.9 %)]. Fever was the most common symptom [*n*=111 (78.2 %)], followed by anorexia [*n*=47 (33.1 %)], abdominal pain [*n*=46 (32.4 %)] and gastrointestinal bleeding [*n*=45 (31.7 %)]. Pathology results showed that the level of procalcitonin was 12.6±29.3 mg l^−1^, and that of C-reactive protein (CRP) was 49.3±70.9 mg l^−1^. The mean level of N-terminal pro-hormone brain natriuretic peptide (NT-proBNP) was 4788.6±4593.8 pg ml^−1^, that of lactate was 3.7±4.6 mmole l^−1^, that of creatinine was 2.31±2.40 mg dl^−1^ and that of albumin was 3.0±0.5 g dl^−1^. Mean activated partial thromboplastin time (APTT) was 47.7±30.3 s. The lowest platelet count was 35 248±43 453 mm^3^, which recovered to 150 891±105 123 mm^3^ before patients were discharged from the ICU. The highest level of aspartate aminotransferase (AST/GOT) was 1133.9±2460.2 IU l^−1^, and of alanine aminotransferase (ALT/GPT) was 398.0±741.9 IU l^−1^. Overall, there were 50 (35.3 %) patients who required mechanical ventilation and 20 (14.1 %) who required continuous renal replacement therapy (CRRT). The mean length of stay (LOS) in the ICU was 7.8±10.3 days and in the hospital was 14.8±16.0 days (Table 4).

**Table 1.** Demographic and clinical variables of dengue groups at admission

Variables	All patients (*n*=142)	Primary bacteraemia (*n*=22)	Control (*n*=120)	P*
Age (years)	69.97±15.93	75.50±7.68	68.95±16.84	0.077
Male patients	70 (49.2 %)	8 (36.4 %)	62 (51.7 %)	0.187
Mortality	32 (22.5 %)	9 (40.9 %)	23 (19.2 %)	0.048
APACHE II score	18.00±9.45	25.18±11.95	16.63±8.51	0.004
TISS	22.87±10.98	28.50±13.15	21.80±10.49	0.010
Glasgow Coma Scale	12.02±4.14	9.64±4.96	12.47±3.90	0.018
Comorbidity	1.74±1.27	2.06±1.00	1.68±1.31	0.220
Hypertension	90 (63.4 %)	17 (77.3 %)	73 (60.8 %)	0.141
Diabetes	70 (49.3 %)	13 (59.1 %)	57 (47.5 %)	0.317
Coronary artery disease	27 (19.0 %)	4 (18.2 %)	23 (19.2 %)	1.000
Cancer	17 (12.0 %)	3 (13.6 %)	14 (11.7 %)	0.729
Stroke	16 (11.3 %)	1 (4.5 %)	15 (12.5 %)	0.467
End-stage renal disease	12 (8.5 %)	4 (18.2)	8 (6.7 %)	0.092
COPD	9 (6.3 %)	2 (9.1 %)	7 (5.8 %)	0.630
Autoimmune disease	3 (2.1 %)	1 (4.5 %)	2 (1.7 %)	0.399
Alcohol	2 (1.4 %)	0 (0.0 %)	2 (1.7 %)	1.000
Liver cirrhosis	1 (.7 %)	0 (0.0 %)	1 (.8 %)	1.000
Multi-organ failures (number of organs)	1.91±1.60	2.68±2.17	1.77±1.44	0.069
Multi-organ failures (number of patients)	126 (88.7 %)	19 (86.4 %)	107 (89.2 %)	0.715
Haematological	112 (78.9 %)	17 (77.3 %)	95 (79.2 %)	0.783
Thoracic	46 (32.4 %)	10 (45.5 %)	36 (30.0 %)	0.154
Cardiovascular	43 (30.3 %)	10 (45.5 %)	33 (27.5 %)	0.092
Renal	31 (21.8 %)	10 (45.5 %)	21 (17.5 %)	0.009
Metabolic acidosis	30 (21.1 %)	8 (36.4 %)	22 (18.3 %)	0.085
Hepatic	14 (9.9 %)	4 (18. %)	10 (8.3 %)	0.233
Mechanical ventilation used	50 (35.3 %)	11 (50.0)	39 (32.5 %)	0.114
CRRT used	20 (14.1 %)	6 (27.3 %)	14 (11.7 %)	0.088

Expressed as mean±sd or frequency (percentage).

APACHE II, Acute Physiology and Chronic Health Evaluation II; TISS, Therapeutic Intervention Scoring System; COPD, chronic obstructive pulmonary disease; CRRT, continuous renal replacement therapy.

*Comparison among dengue patients with and without primary bacteraemia.

**Table 2.** Laboratory data of dengue groups at admission

Variables	All patients (*n*=142)	Primary bacteraemia (*n*=22)	Controls (*n*=120)	P*
Procalcitonin, ng ml^−1^	12.55±29.34	36.90±65.79	8.08±11.23	0.053
C-reactive protein, mg l^−1^	49.27±70.86	119.04±130.90	36.48±43.13	0.008
NT-proBNP, pg ml^−1^	4788.46±4593.83	4325.37±4150.20	4873.36±4681.70	0.609
APTT (s)	47.67±30.26	66.54±48.91	44.21±24.18	0.047
Diagnostic tests				
NS1	114 (80.3 %)	17 (77.3 %)	97 (80.3 %)	0.700
IgM	39 (27.5 %)	4 (18.2 %)	35 (29.2 %)	0.289
IgG	36 (25.4 %)	5 (22.7 %)	31 (25.8 %)	0.758
PCR	37 (26.8 %)	6 (27.3 %)	31 (26.7 %)	0.958
BUN, mg dl^−1^	35.82±29.04	46.19±30.77	33.92±28.44	0.068
Creatinine, mg dl^−1^	2.31±2.40	2.58±2.07	2.26±2.46	0.563
Sodium, mmol l^−1^	134.00±5.52	134.62±6.13	133.89±5.42	0.569
Potassium, mmol l^−1^	3.96±.97	4.12±1.58	3.93±.82	0.389
Lactate, mmol l^−1^	3.74±4.59	6.15±7.13	3.30±3.84	0.080
I/O>5000 after ICU 3 days	35 (24.6 %)	12 (54.5 %)	23 (19.2 %)	<0.001
Lactate >2 after ICU 3 days	40 (28.2 %)	12 (54.5 %)	28 (23.3 %)	0.003
Albumin, g dl^−1^	3.02±.52	2.76±.55	3.07±.51	0.011
Haemoglobin, g dl^−1^	12.30±3.11	11.75±2.73	12.40±3.18	0.363
Haematocrit, %	35.46±8.50	33.85±7.66	35.75±8.64	0.337
Haematocrit change >20 % after ICU 3 days	35 (24.6 %)	8 (36.4 %)	27 (22.5 %)	0.165
Platelets on admission, 10^3^ mm^−3^	97 626.06±84 919.61	105 909.09±85 508.51	96 107.50±85 083.60	0.620
Lowest platelet count, 10^3^ mm^−3^	35 248.61±43 453.60	33 590.91±40 332.73	35 553.52±44 154.57	0.846
Last platelet count before ICU discharge, 10^3^ mm^−3^	150 891.92±105 123.65	124 005.93±110680.38	155 821.01±103 798.33	0.193
Leukocyte count, 10^3^ mm^−3^	8614.70±13 965.26	14 359.09±17 431.60	7561.67±13 047.72	0.035
Highest AST/GOT, IU l^−1^	1133.89±2460.23	1665.55±3223.72	1036.41±2296.94	0.390
Highest ALT/GPT, IU l^−1^	398.03±741.86	674.68±1230.85	347.32±606.64	0.235

Expressed as mean±sd and frequency (percentage).

NT-proBNP, N-terminal of the pro-hormone brain natriuretic peptide; BUN, blood urea nitrogen; AST/GOT, aspartate aminotransferase; ALT/GPT, alanine aminotransferase; APTT, activated partial thromboplastin time; PCR, polymerase chain reaction; NS1, non-structural protein 1.

*Comparison among dengue patients with and without concurrent bacteraemia.

**Table 3.** Symptoms among dengue groups at admission

Variables	All patients (*n*=142)	Primary bacteraemia (*n*=22)	Controls (*n*=120)	P*
Symptoms	2.30±1.48	1.95±1.46	2.36±1.48	0.241
Fever	111 (78.2 %)	19 (86.4 %)	92 (76.7 %)	0.407
Anorexia	47 (33.1 %)	6 (27.3 %)	41 (34.2 %)	0.528
Abdominal pain	46 (32.4 %)	3 (13.6 %)	43 (35.8 %)	0.041
Gastrointestinal bleeding	45 (31.7 %)	8 (36.4 %)	37 (30.8 %)	0.608
Myalgia	25 (17.6 %)	3 (13.6 %)	22 (18.3 %)	0.766
Haematuria	26 (18.3 %)	2 (9.1 %)	24 (20.0 %)	0.368
Diarrhoea	13 (9.2 %)	1 (4.5 %)	12 (10.0 %)	0.692
Skin rashes	10 (7.0 %)	0 (0.0 %)	10 (8.3 %)	0.361
Gum bleeding	3 (2.1 %)	1 (4.5 %)	2 (1.7 %)	0.399

Expressed as mean±sd or frequency (percentage).

*Comparison among dengue patients with and without primary bacteraemia.

**Table 4.** Clinical outcome for dengue groups

Variables	All patients (*n*=142)	Primary bacteraemia (*n*=22)	Controls (*n*=120)	P*
ICU stay (days)	7.84±10.28	4.23±2.47	8.50±11.02	0.073
Hospital stay (days)	14.79±16.02	12.49±16.28	15.21±16.01	0.465
Medical expenses (NT$1000)	182.20±260.52	124.86±81.96	192.71±280.21	0.263
Hospital mortality [*n* (%)]	31 (21.8 %)	9 (40.9 %)	22 (18.3 %)	0.018

Expressed as mean±sd frequency (percentage).

*Comparison among dengue patients with and without primary bacteraemia.

Twenty-two patients (15.5 %) had concurrent bacteraemia; species of the genus *Streptococcus* were the most common pathogen [*n*=6 (27.3 %)], followed by *Escherichia coli* [*n*=5 (22.7 %)], species of the genus *Staphylococcus* [*n*=3 (13.6 %)], species of the genus *Micrococcus* [*n*=2 (9.1 %)] and one patient each with *Pseudomonas aeruginosa*, *Acintobacter baumannii*, *Klebsiella pneumoniae*, a species of the genus *Proteus*, a species of the genus *Moraxella* and a species of the genus *Ralstonia*. Patients with bacteraemia had higher APACHE II and TISS scores, CRP levels, leukocyte counts, positive fluid balances, longer APTTs, more kidney failure and elevated lactate levels, but lower GCS scores and albumin levels and less abdominal pain than did patients in the control group without concurrent bacteraemia (all *P*<0.05) (Tables 1, 2 and 3). Additionally, patients with bacteraemia had a significantly (*P*=0.018) higher mortality rate than did the control group (40.9 % vs 18.3 %) (Table 4). Multiple logistic regression analysis results indicated that bacteraemia was significantly positively associated with the following independent predictors: higher CRP level [adjusted odds ratio (aOR): 1.026; 95 % confidence interval (CI): 1.008–1.044; *P*=0.005] and longer APTT (aOR: 1.034; 95 CI: 1.004–1.065; *P*=0.027) (Table 5).

**Table 5.** Independent risk factors for concurrent primary bacteraemia in patients with dengue at admission

Variables*	Adjusted OR	HR 95 % CI		*P*
		Lower	Upper	
Age (years)	1.110	0.981	1.257	0.098
Gender (male)	0.207	0.033	1.289	0.091
C-reactive protein (mg l^−1^)	1.026	1.008	1.044	0.005
APTT (s)	1.034	1.004	1.065	0.027
Highest AST/GOT (IU l^−1^)	0.999	0.998	1.000	0.096

Conditional logistic regression modelling was performed (only *P*<0.100 is presented).

APTT, activated partial throboplastin time; OR, odds ratio; CI, confidence interval.

*Age, gender and highest AST/GOT were marginally associated with the risk of primary bacteraemia infections (*P*<0.100).

Although patients with concurrent bacteraemia and dengue infections tended to have higher ICU and in-hospital mortality than did matched controls with bacteraemia only, the difference was not significant (Table 6). However, the bacteraemia without dengue group had longer ICU stays and higher medical expenses than did patients with concurrent bacteraemia and dengue.

**Table 6.** Demographic and clinical outcome of the bacteraemia dengue group and bacteraemia without dengue (control) group over the same period

Variables	Bacteraemia with dengue infection (*n*=22)	Bacteraemia without dengue infection (*n*=44)	*P*
Age (years)	75.50±7.68	75.53±7.68	0.913
Male patients	8 (36.4 %)	16 (36.4 %)	1.000
APACHE II score	25.18±11.95	26.48±8.00	0.649
TISS	28.50±13.15	28.45±8.69	0.988
Glasgow Coma Scale	9.64±4.96	8.89±3.24	0.525
Gram-positive organism	14 (63.6 %)	15 (34.1 %)	0.035
Gram-negative organism	10 (45.5 %)	29 (65.9 %)	0.111
ICU stay (days)	4.23±2.47	9.57±8.02	<0.001
Hospital stay (days)	12.49±16.28	15.20±8.40	0.375
Medical expenses (NT$1000)	124.86±81.96	249.29±219.08	<0.001
ICU mortality	9 (40.9 %)	10 (22.7 %)	0.124
Hospital mortality	9 (40.9 %)	12 (27.3 %)	0.262

Expressed as mean±sd and frequency (percentage).

APACHE II, Acute Physiology and Chronic Health Evaluation II; TISS, Therapeutic Intervention Scoring System; COPD, chronic obstructive pulmonary disease; CRRT, continuous renal replacement therapy.

## Discussion

This is the first study, to our knowledge, that, in order to predict which patients have concurrent bacteraemia, focused on those with a severe dengue infection and who required ICU admission. We have several significant findings.

First, we found that 22 (15.5 %) of the patients with severe dengue infection and who required admission to the ICU had bacteraemia. This finding is different from those of Lee *et al.* (2005) and Thein *et al.* (2015). In the former study, seven (5.5 %) of 127 patients with dengue haemorrhagic fever (DHF) or dengue shock syndrome (DSS) had concurrent bacteraemia. In the latter study, only 18 (0.2 %) of 9553 patients had concurrent bacteraemia. However, our findings are consistent with those of Leo *et al.* (2011), who reported that four (14.3 %) of 27 patients who died because of dengue had bacteraemia. The differences between these studies might be because of the different levels of severity used to analyse dengue-infected patients in each study (Lee *et al.*, 2005; Leo *et al.*, 2011; Thein *et al.*, 2015). Our study and that of Leo *et al.* (2011) included only patients who required admission to the ICU or who died because of a dengue infection; thus, both studies had a higher rate of concurrent bacteraemia. In contrast, there were only 26 patients severely affected by dengue in Thein *et al.* (2015). Overall, these findings suggest that the rate of bacteraemia might increase with the severity of dengue infection. In critically ill patients with dengue infection, clinicians must be aware of the possibility of concomitant bacteraemia.

Second, we found that dengue-infected patients with concurrent bacteraemia had a higher mortality rate than did the control group. This finding is consistent with that of Lee *et al.* (2005), who reported that the mortality rate of patients with dual infection was higher than that of patients with DHF or DSS alone (28.5 vs 1.1; *P*=0.012). Two other mortality studies on dengue infection (Ong *et al.*, 2007; Lahiri *et al.*, 2008) also reported that bacteraemia was associated with 42.9 and 44.4 %, respectively, of dengue-related deaths. All of these findings should indicate the importance of an early diagnosis of concomitant bacteraemia and prompt antibiotic treatment for critically ill patients with a dengue infection.

In addition, to determine whether the bacterial infections were solely responsible for increased mortality, we also provided a group of matched controls with bacteraemia but not dengue infection who were admitted to the ICU during the same period. In theory, a severe dengue infection *per se* perhaps also contributed to increased mortality. Although the patients with dengue and bacterial co-infections had significantly lower ICU stays and medical expenses, their higher trends of ICU and in-hospital mortality than the controls with bacteraemia but without a dengue infection indicated that a bacterial infection does significantly exacerbate dengue infection, and a severe dengue infection also increases the risk that a patient with bacteraemia will die of the disease. The relatively lower ICU stays but higher mortality in patients with severe dengue and bacteraemia co-infections might hint at death rather than recovery compared with patients who have a severe dengue infection or bacteraemia alone.

Third, the most common pathogen in the 22 cases of bacteraemia in this study was species of the genus *Streptococcus*, followed by *Escherichia coli* and then species of the genus *Staphylococcus*. However, in Lee *et al.* (2005), of seven cases with concurrent DHF or DSS and bacteraemia, *Klebsiella pneumoniae* was the most common pathogen (*n*=3), followed by one species each of a species of the genus *Rosemonas*, *Moraxella lacunata*, *Klebsiella ozaenae* and *Enterococcus faecalis*. In Thein *et al.* (2015), *Staphylococcus aureus* was the most common pathogen, followed by *Salmonella typhi.* In Leo *et al.* (2011), of four deaths in patients with bacteraemia and dengue, two were caused by methicillin-resistant *S. aureus* (MRSA), and one each was caused by *P. aeruginosa* and alpha-haemolytic *Streptococcus*. These findings indicate that the microbiological distribution of dengue infection with concomitant bacteraemia varied in each cited study. However, because the number of infections in each study is small, the studies are likely to be affected by stochastic sampling. Overall, empirical broad-spectrum antibiotic therapy should be used first, and then it should be switched to a specific antibiotic therapy that is based on final culture results and is appropriate for the patient’s clinical condition.

Finally, we found that the clinical manifestations of patients with severe dengue and bacteraemia–APACHE II, TISS, and GCS scores; leukocyte counts; fluid balance; APTT; kidney failure; CRP, lactate and albumin levels; and frequency of abdominal pain–were significantly different from those of patients without bacteraemia. Lee *et al.* (2005) and Thein *et al.* (2015) identified several risk factors for bacteraemia in dengue patients: acute renal failure, prolonged fever and a Pitt bacteraemia score ≥4. Most importantly, we found that higher CRP levels and longer APTT were two independent risk factors, which might be useful for predicting concurrent bacteraemia in patients severely affected by dengue.

Our study has some limitations. First, the number of cases is limited and the present work is a single-center study. Therefore, our findings might not be generalizable to other hospitals. Additional large-scale investigations are warranted. Second, because our laboratory cannot assess the serotype of dengue virus, we have no information about the type of dengue fever in the individual patient.

In conclusion, concomitant bacteraemia is not uncommon in patients severely affected by dengue and who require admission to the ICU, and it is associated with high mortality. Additionally, higher CRP and longer APTT are two independent risk factors associated with concurrent bacteraemia. All of our findings indicate that intensivists should be alert to the possible occurrence of concurrent bacteraemia, which is a clinical entity of substantially high mortality among severely ill dengue patients who require admission to the ICU, especially for those with high CRP levels or prolonged APTT.

## Supplementary Data

276 Supplementary File 1
