# Supplementary material for: The exploration of risk factors of concurrent bacteraemia in patients critically ill with severe dengue
Source: J Med Microbiol. 2016 Dec 16;65(12):1505–11. doi: 10.1099/jmm.0.000388 (PMC5203669; doi:10.1099/jmm.0.000388)
Supplement: Supplementary File 1 [file jmm-65-1505-s001.pdf]

Appendix 1: Definitions of organ failures

1. Hematologic failure: a platelet count < 80,000/mm^3 or a 50% decrease in the platelet count from the highest value recorded over the previous 3 days.
2. Thoracic failure: a ratio < 200 of the partial pressure of oxygen in the patient's arterial blood to the fraction of oxygen in the inspired air.
3. Cardiovascular failure: systolic blood pressure (SBP) of ≤ 90 mmHg; a mean arterial pressure (MAP) ≤ 65 mmHg for at least 1 hour despite adequate fluid resuscitation; or the need for vasoactive agents (dopamine ≥ 5 mg/kg/min) to maintain SBP ≥ 90 mmHg or MAP ≥ 65 mmHg.
4. Kidney failure: a creatinine level ≥ 2 mg/dL, or oliguria with an average urine output < 0.5 mL/kg/h for 4 hours despite adequate fluid resuscitation
5. Metabolic acidosis (failure): a pH ≤ 7.30, or a base deficit ≥ 5.0 mEq/L and a plasma lactate level > 3 mmol/L.
6. Hepatic failure: a serum bilirubin level markedly higher than ≥ 4 mg/dL.
